# An uncommon cause of tardy ulnar nerve palsy due to upper extremity prolonged malposition in a comatose child: a case report

**Published:** 2012-11

**Authors:** Mohammad Reza Emamhadi, Davood Mahmoudi

**Affiliations:** ^a^Department of Neurosurgery Peripheral Nerve Center of Brachial Plexus , Gilan University of Medical Sciences, Gilan, Iran.

## Abstract

**Background:**

Ulnar nerve neuropathy is one of the most common peripheral nerve dysfunctions. Elbow is the most common area affected by ulnar nerve which is mainly because of fractures or dislocations of this area. Delayed ulnar nerve palsy (Tardy Ulnar Nerve Palsy) in children due to a malpositioning of upper extremity during hospitalization is an uncommon cause of ulnar nerve injury which we have already reported it.

**Methods:**

An eight-year-old conscious patient who had weakness, paresthesia and tingling in the right 4th and 5th fingers, as well as right claw hand deformity was evaluated, he had attended once before in 4 months ago due to head trauma in coma state. The child had no clinical and radiological indications of arm or elbow fractures causing nerve compression or entrapment. Elbow malposition had caused ulnar nerve neuropathy during hospitalization. Surgery was attempted, ulnar nerve decompression and anterior transposition done.

**Results:**

After three weeks post operatively, active physical therapy was started on the right upper extremity and the hand returned to normal activity after 6 months.

**Conclusions:**

In patients with decreased level of consciousness or coma state who need prolonged hospitalization, the limbs must remain in correct position to prevent superficial nerve injuries and neuropathies. Furthermore, careful and scrutinized attention to the traumatic patients and doing on time and targeted imaging, regular follow up of patients, complete and perfect neurological examinations can prevent peripheral nerve injuries or develop on-time treatments which improve the patients' quality of life.

**Keywords:**

Ulnar nerve, Elbow malposition, Ulnar nerve decompression


					Volume 4, Suppl. 1 Nov 2012
Publisher: Kermanshah University of Medical Sciences
URL: http://www.jivresearch.org
Copyright:
Congress of Iranian Neurosurgeons, 14-16 November 2012, Kermanshah, Iran
Paper No. 62
An uncommon cause of tardy ulnar nerve palsy due to upper
extremity prolonged malposition in a comatose child: a case
report
Mohammad Reza Emamhadi a
, Davood Mahmoudi a,*
a
Department of Neurosurgery, Center of Peripheral Nerve and Brachial Plexus , Gilan University of Medical Sciences, Gilan,
Iran.
Abstract:
Background: Ulnar nerve neuropathy is one of the most common peripheral nerve dysfunctions. Elbow is the most
common area affected by ulnar nerve which is mainly because of fractures or dislocations of this area. Delayed ulnar
nerve palsy (Tardy Ulnar Nerve Palsy) in children due to a malpositioning of upper extremity during hospitalization
is an uncommon cause of ulnar nerve injury which we have already reported it.
Methods: An eight-year-old conscious patient who had weakness, paresthesia and tingling in the right 4th and 5th
fingers, as well as right claw hand deformity was evaluated, he had attended once before in 4 months ago due to
head trauma in coma state. The child had no clinical and radiological indications of arm or elbow fractures causing
nerve compression or entrapment. Elbow malposition had caused ulnar nerve neuropathy during hospitalization.
Surgery was attempted, ulnar nerve decompression and anterior transposition done.
Results: After three weeks post operatively, active physical therapy was started on the right upper extremity and
the hand returned to normal activity after 6 months.
Conclusions: In patients with decreased level of consciousness or coma state who need prolonged hospitalization, the
limbs must remain in correct position to prevent superficial nerve injuries and neuropathies. Furthermore, careful and
scrutinized attention to the traumatic patients and doing on time and targeted imaging, regular follow up of patients,
complete and perfect neurological examinations can prevent peripheral nerve injuries or develop on-time treatments
which improve the patients' quality of life.
Keywords:
Ulnar nerve, Elbow malposition, Ulnar nerve decompression
* Corresponding Author at:
Davood Mahmoudi: Department of Neurosurgery Peripheral Nerve and Brachial Plexus Center, Guilan University of Medical Sciences, Guilan,
Iran, Email: drdmahmoudi@yahoo.com , (Mahmoudi D.).

				

